# A Cellular Automata-Based Mathematical Model for Thymocyte Development

**DOI:** 10.1371/journal.pone.0008233

**Published:** 2009-12-09

**Authors:** Hallan Souza-e-Silva, Wilson Savino, Raúl A. Feijóo, Ana Tereza Ribeiro Vasconcelos

**Affiliations:** 1 Bioinformatics Laboratory, LNCC/MCT, Petrópolis, Brazil; 2 Laboratory of Thymus Research, Oswaldo Cruz Institute, Fiocruz, Rio de Janeiro, Brazil; 3 Computer Science Coordination, LNCC/MCT, Petrópolis, Brazil; 4 National Institute of Science and Technology for Medicine Assisted by Scientific Computing, INCT-MACC, Petrópolis, Brazil; University of Swansea, United Kingdom

## Abstract

Intrathymic T cell development is an important process necessary for the normal formation of cell-mediated immune responses. Importantly, such a process depends on interactions of developing thymocytes with cellular and extracellular elements of the thymic microenvironment. Additionally, it includes a series of oriented and tunely regulated migration events, ultimately allowing mature cells to cross endothelial barriers and leave the organ. Herein we built a cellular automata-based mathematical model for thymocyte migration and development. The rules comprised in this model take into account the main stages of thymocyte development, two-dimensional sections of the normal thymic microenvironmental network, as well as the chemokines involved in intrathymic cell migration. Parameters of our computer simulations with further adjusted to results derived from previous experimental data using sub-lethally irradiated mice, in which thymus recovery can be evaluated. The model fitted with the increasing numbers of each CD4/CD8-defined thymocyte subset. It was further validated since it fitted with the times of permanence experimentally ascertained in each CD4/CD8-defined differentiation stage. Importantly, correlations using the whole mean volume of young normal adult mice revealed that the numbers of cells generated in silico with the mathematical model fall within the range of total thymocyte numbers seen in these animals. Furthermore, simulations made with a human thymic epithelial network using the same mathematical model generated similar profiles for temporal evolution of thymocyte developmental stages. Lastly, we provided *in silico* evidence that the thymus architecture is important in the thymocyte development, since changes in the epithelial network result in different theoretical profiles for T cell development/migration. This model likely can be used to predict thymocyte evolution following therapeutic strategies designed for recovery of the thymus in diseases coursing with thymus involution, such as some primary immunodeficiencies, acute infections, and malnutrition.

## Introduction

T lymphocytes play an important role in the adaptive immune response and their development is fundamental in the process of defense against pathogens as well as tumor cells. These lymphocytes are able to provide immune memory and to respond to hundreds of thousands of different pathogens through specific T cell receptors (TCR) [1]. The development of T cells occurs in the thymus and is the process by which bone marrow-derived precursor cells mature and gain T cell receptors, being able to mount and participate in cell-mediated immune responses.

Intrathymic T cell differentiation is a complex sequence of biological events, comprising cell proliferation, differential membrane protein expression, gene rearrangements, and massive programmed cell death [2]. The normal T cell development is important in generating and regulating efficient cell-mediated immune responses in the peripheral lymphoid organs and sites of effector activities. The thymus provides a specialized and architecturally organized microenvironment for T cell development and consists of numerous lobules, each one differentiated into an outer cortical region and an inner medulla. Migration through the thymic lobule is crucial for thymocyte development, and is influenced by chemokines and by interactions with a network of microenvironmental cells, whose major component is the epithelium [2]–[4]. This migratory process occurs in a spatially and chronologically well-defined manner that can be summarized as follows: 1) the entry of bone marrow-derived double negative (DN) (CD4^−^CD8^−^) progenitors into the thymus by cortex-medulla junction (CMJ); 2) migration of immature DN cells, from the CMJ towards the subcapsular zone (SCZ) of the thymic lobule, with subsequent proliferation and differentiation of double positive (DP) (CD4^+^CD8^+^) cells; 3) reorientation of the migratory process, now towards the medulla, positive selection, and generation of single positive (SP, CD4^+^CD8^−^ or CD4^−^CD8^+^) cells; 4) further interactions of SP thymocytes with epithelial and dendritic cells in the medulla for complete development (negative selection) to ensure central tolerance; and 5) egress of mature SP T cells to peripheral lymphoid organs.

At the end of the differentiation route, cells are committed to the T lymphocyte lineages CD4 or CD8. The moment and the exact mechanism of intrathymic lineage commitment, as well as the mechanisms leading to a 2∶1 CD4/CD8 T cell ratio in the thymus remain unknown. Signals elicited by binding of the T cell receptor (TCR) and the CD4/CD8 co-receptor to type I or type II major histocompatibility (MHC)/peptide complex determine the positive and negative selection events, as well as the control of commitment towards the differentiation into CD4 or CD8 single positive cells. It has been shown that a Notch receptor [5], [6] and the duration of the TCR signal are involved with CD4 *versus* CD8 lineage commitment [7]. In this case, the “*signal-duration” model* postulates that long duration TCR signals instruct DP thymocytes to differentiate in CD4SP T cells and short duration TCR signals instruct DP thymocytes to differentiate in CD8SP T cells [7], [8].

Interactions between thymocytes and the thymic microenvironment are bi-directional: If on one hand cell-cell interactions determine the fate of developing thymocytes, on the other hand they also control the architecture of thymic microenvironments, thus characterizing a “crosstalk” between the lymphoid and the microenvironmental compartments within the organ [9].

In recent years, mathematical modeling and computational simulation assumed an important role in the sense of completing the experimental modeling to study a variety of biological events, including those discussed above. In this respect, mathematical modeling has been applied to study some specific issues related to T cell development, as for example, a) to compare the intrathymic development of bone marrow precursors, derived either from young or old donors [10]; b) to evaluate thymic egressors, using the T cell receptor excision circle (TREC) concept [11]; and c) to approach the entire development of thymocyte subsets [12]–[15].

The mathematical model used to study the effect of aging on bone marrow cells colonizing the thymus [10] applied ordinary differential equations (ODE) to describe the system. In this model, equations did not discriminate among thymocyte subsets and showed only the evolution time of proliferating and resting cell populations in the thymus, after a single wave of seeding bone marrow cells. Actually, the model was applied to test hypotheses and analyze the mechanism underlying the observed developmental inferiority of bone marrow-derived thymic progenitors from old mice when competing with bone marrow progenitors from young mice [16]. The relevance of such evaluation is that the knowledge related to the effect of aging on bone marrow cells can help in understanding the decrease in efficiency of the immune system in the elderly, which is also related to the physiological age-dependent thymic involution. In this context, the model revealed that none of the parameters evaluated – time of cell-cycle, rates of change to resting state, and rates of cell death – was in itself sufficient to explain the different behavior of the thymocytes derived from old *versus* young bone marrow, leading to the notion that further experiments should be performed to better tackle this issue [10].

A second mathematical approach, also applying ordinary differential equations, focused on the naïve T cell compartment (corresponding to the recent thymic emigrants) defined by the presence of TREC formed during T cell receptor gene rearrangement [11]. The authors combined mathematical modeling with TREC measurements and cell division rates in naïve T cells of healthy and HIV-1 infected individuals, in order to obtain a better interpretation of TREC measurements in respect to thymic output. The mathematical equations described the time evolution of the naïve T cells and the total number of TRECs, and showed, among other results, that naïve T cell division would have a rapid and strong effect on the TREC average [11].

The first mathematical model to study thymocyte subset dynamics was introduced by Ramit Mehr and co-workers, aiming to capture the essential features of T cell differentiation and selection [12]. In this model the authors applied a set of ordinary differential equations to define time evolution of thymocyte subsets, including DN, DP, CD4SP, and CD8SP cells. The model reproduces the percentages of thymocyte subpopulations as seen in biological samples and predicts that negative selection likely operates at the DP stage or later. Other results of the model revealed that the CD4SP over CD8SP cell ratio fits the “*instructive*” theory of thymic lineage commitment [17], [18].

As regards thymocyte subset dynamics, a single experimental system of transient perturbation of T lymphocyte homeostasis was evaluated in combination with a mathematical model, to quantify dynamics of mouse T cell differentiation [15], which took into account the concept by which thymocyte migrate as if they were on a “conveyor belt” [19]. By using differential equations, cell numbers at each division stage within different T cell compartments (from DN thymocytes to naïve T cells) in the spleen were monitored at different time points during and after ganciclovir treatment. The mathematical model was based on a “conveyor-belt” [19] type of differentiation and allowed the study of cell fluxes, residence times, and rates of import, export, proliferation, and death across cell compartments for thymocytes and recent thymic emigrants.

The mathematical model introduced by Sol Efroni and co-workers was different in that it also took into account the spatial information in thymocyte development, being based on reactive animation [20]. In this model, twelve thymocyte subsets were not treated with differential equations, but in a discrete way and were represented by differential states. For example, a given cell may be described as expressing several receptors, no receptors at all, or any combination of receptors at different stages of the cell cycle and in specific anatomical compartments [21]. With this model it was possible to describe the developmental stage and positioning of each cell in the system, whereas with the ODE discussed above, only the global information of each thymocyte subset could be measured. In their differentiation route, the cells placed in the reactive animation model moved following gradients of concentration and interacted with epithelial cells in distinct thymic microenvironments. In this model, cell migration patterns were in agreement with experimental results performed with CXCR4^−/−^ and CCR9^−/−^ knockout mice. The model also predicted that competition between thymocytes for interaction space and cell dissociation rate would determine the T cell development and the lineage commitment to CD4 or CD8 [18].

Overall, the data summarized above indicate that the use of mathematical models in the study of the mechanisms governing thymocyte migration may provide new clues for designing therapeutic strategies targeting T cell precursors and to predict abnormalities of such process.

We introduced herein a mathematical model based on cellular automata [22] to describe thymocyte migration and development within the thymus. One of the main characteristics of cellular automata is the appearance of global properties, which in turn emerge from the local interactions and that hardly would be seen from the information of the basic elements of the system, where the rules of the model are defined [22], being quite useful to study complex systems [23]. Our initial model takes into account the main cells involved in the lympho-microenvironmental interactions: double negative (CD4^−^CD8^−^), double positive (CD4^+^CD8^+^) and single positive (CD4^+^CD8^−^ and CD4^−^CD8^+^) thymocytes, as well as epithelial cells, and the influence of chemokines in the cell migration. This model, different from the others discussed above, uses in the simulations, epithelial networks obtained from experimental data (derived from immunolabeling of thymus frozen sections with antibodies specific to the thymic epithelial cell network) to induce thymocyte differentiation and migration.

## Results

The parameters of our mathematical model, described in details in the *Materials and Methods* section, as well as the values adopted in the simulations, are summarized in the Table 1. The values were ultimately determined by adjusting the pre-designed model to the experimental results previously obtained with mice, in which thymocyte development was evaluated during thymus recovery following sub-lethal γ-ray irradiation [24]. Should we point out that the values of some parameters shown in Table 1 can effectively change depending on the mathematical rule of evolution and on the dynamics of the system. This comprises thymocyte speed; rate of cell entry into the thymus, as well as the division rates of DN and of DP cells. For example, 0.18 division/min occurs in the beginning of DN expansion, but after the steady state is achieved, the model finds 13 divisions per day.

**Table 1:** Parameters of the mathematical model for thymocyte migration and differentiation*.

Parameter	Value	Parameter Definition
*S*	∼2.54 *µm*/min	thymocyte speed
*T_entry_*	∼4.*1×*10*^−^* ^3^ cells/min	rate of entry of thymocytes
*N_max_*	1024 cells	maximum number of cells generated by division
*T_DIV_DN_*	0.18 div/min	rate of replication to DN cell
*T_DP_*	∼1.*9×*10*^−^* ^3^ cells/min	rate of differentiation DN to DP
*T_DIV_DP_*	∼0.062 div/min	rate of replication to DP cell
*p_mov_*	0.66	additional probability of motion for a site occupied by a network epithelial
*N*_DP_	150 interactions	number of interaction of DP cell before positive selection or apoptose (avidity)
*θ*_1_ DP cells	32	low affinity threshold of TCR DP cell
*θ*_2_ DP cells	87	high affinity threshold of TCR DP cell
*T_C_*	1.53	CD4 fate threshold
*T_ap_*	∼5.6 days	time to apoptosis due to lack of stimuli
*N*_SP_	100 interactions	number of interaction of SP cell before positive selection or apoptose (avidity)
*θ*_1_ SP cells	30	low affinity threshold of TCR SP cell
*θ*_2_ SP cells	60	high affinity threshold of TCR SP cell
*T_int_*	∼24.2 minutes	Interaction time between a thymocyte and the epithelial network
*K*	1 unit	amount of chemokine produced by epithelial network in a site/lattice update
*D*	0.05	chemokine degradation in a site/lattice update

The value of the parameters were ultimately determined after fitting the model with experimental results [24]. The values of some parameters (thymocyte speed; rate of cell entry into the thymus, division rate of DN and of DP cells), can change depending on the mathematical rule of evolution and on the dynamic of the system. For example, 0.18 division/min occurs in the beginning of DN expansion, but after the steady state is achieved, the model finds 13 divisions per day.

* Each lattice update is equivalent to ∼1.61 minutes.

### General Characteristics of the Mathematical Model, as Applied to the Mouse Thymus

**Figure 1:**
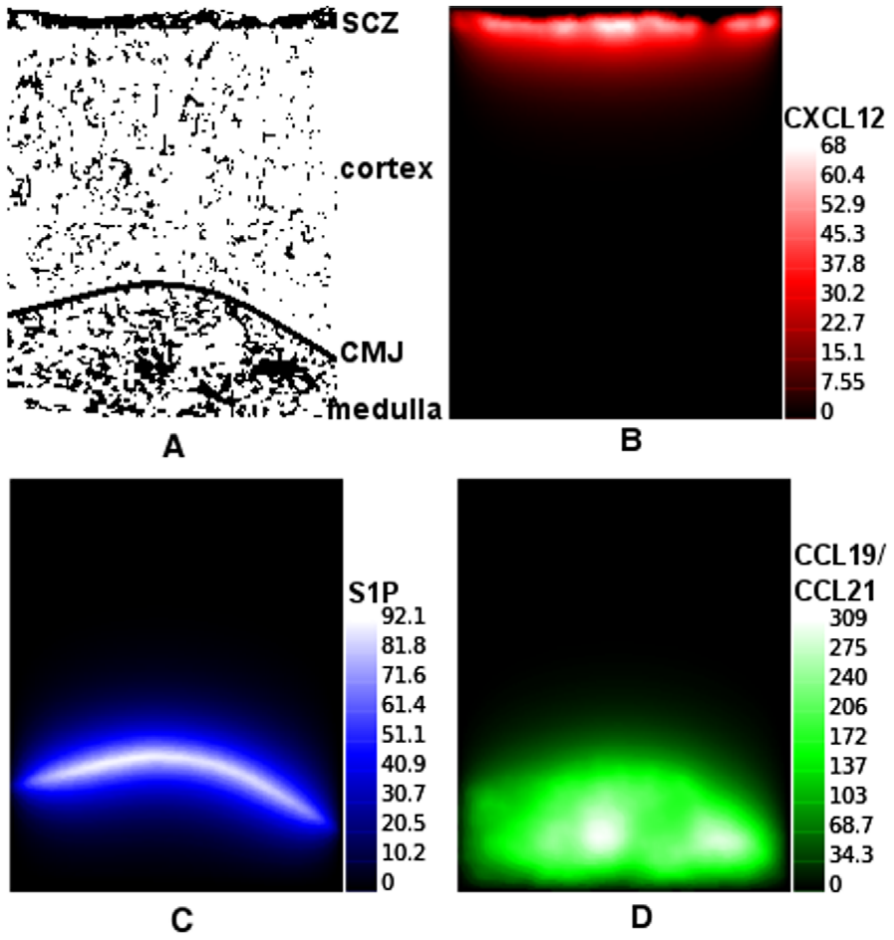
Spatial distribution of chemokines in the thymic lattice obtained with the mathematical simulations. Panels show the distribution of chemokines within the thymic lattice, during the steady state. The painel (A) shows the epithelial network, herein represented as the source of chemokines. Concentrations of the various attractive molecules are shown in panels (B), (C), and (D), respectively for CXCL12, S1P, and CCL19/CCL21. The unit of chemokines provided herein is arbitrary.

We first applied the our cellular automata model to approach the production and the diffusion of chemokines in the digital lattice, including the spatial gradient responsible for thymocyte migration. The relevance of the study with chemokines lies on the fact that it can help us to determine relevant molecules involved in thymocyte migration, in respect to each stage of thymocyte development. The spatial distribution for each chemokine depends of the epithelial network used Fig. 1A (see too the Material and Methods section) and has largest concentrations located in specific regions: SCZ for CXCL12, CMJ for S1P, and medulla for CCL19 and CCL21 (Fig. 1B–D). In the medulla, for example, we can easily observe two large clusters (adjacent sites) overlapped by the epithelial network (Fig. 1D), therefore with large quantities of chemokines being produced in these regions. The unit for chemokines is arbitrary, and in the present model the chemokines essentially serve to guide the movement of thymocytes from a region of lower concentration to the highest concentration.

**Figure 2:**
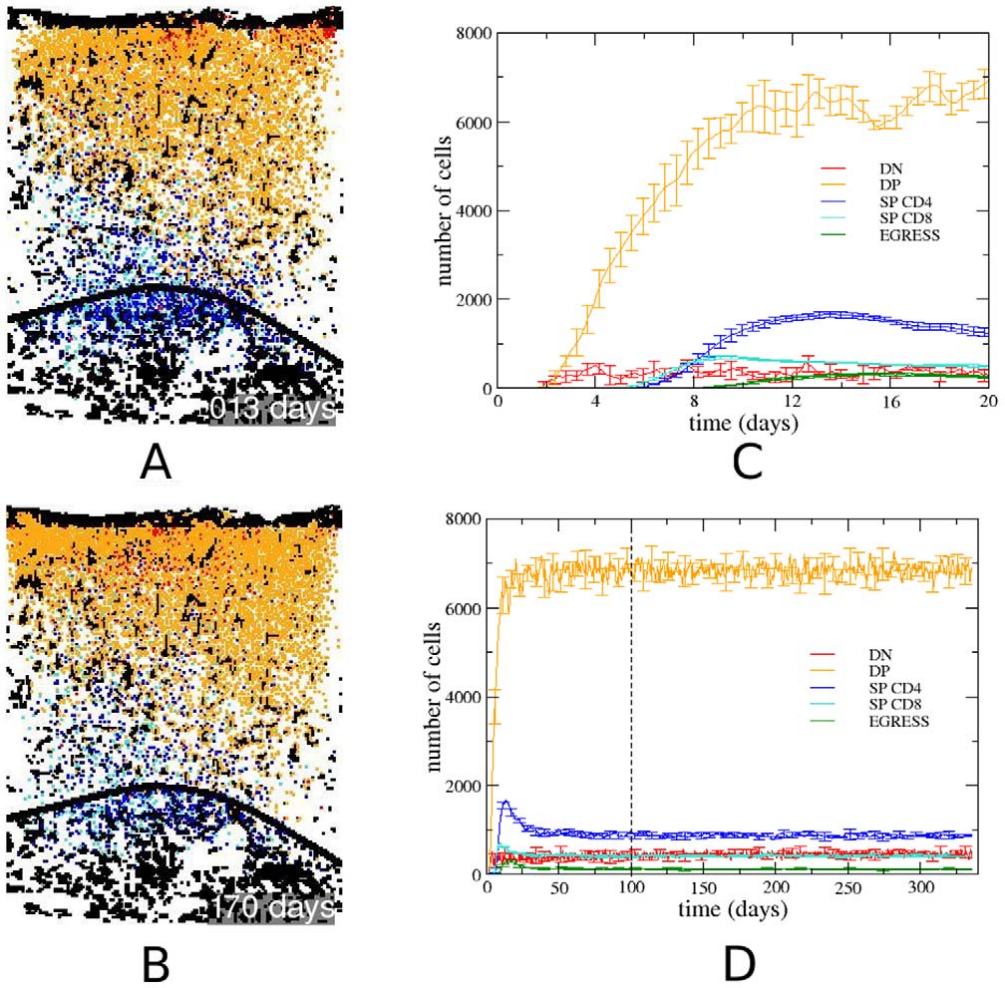
Pattern of thymocyte distribution, development and temporal evolution in the mouse thymic epithelial network. Temporal evolution of thymocyte migration and development in the lattice. Frames with (A) ∼10 days, (B) ∼170 days. Legend of colors: black for the epithelial network, red for DN cells, orange for DP cells, blue for CD4SP cells, and cyan for CD8SP cells. The black color is shown for the site with a thymocyte and a portion of the epithelial network. The temporal evolution of these thymocyte subsets can be seen in panels (C) and (D), where the vertical dashed line indicates the moment the system reaches the steady state. The (C) and (D) panel also depicts the curve of cells egressing the organ.

With the automata cell model we can observe the migration and development of a given thymocyte from the initial stage, where only DN cells exist in the lattice, until a steady state where we find thymocytes in all stages of development adopted in the model (Fig. 2) (see the Movie S1). The thymocytes in different stages of development are predominantly in specific regions of the lattice – DN cells in the subcapsular zone, DP cells in the cortex, and SP cells in the medulla. These results are in agreement with the spatial distribution observed experimentally in mice [2].

According to our model, in normal conditions the first cell divisions occur at ∼2 days, when the DN cells, guided by CXCL12 gradients (Fig. 1B) reach the subcapsular zone. Then, at ∼4.5 days, DN cells that differentiated in DP cells are, in SCZ, in migration towards the medulla following CCL19/CCL21 gradients (Fig. 1D). As previously reported [14], by this time a spatial competition exists between the DN cells in migrating to SCZ, and the DP cells that migrate towards to medulla. Theoretically, this competition can delay the process of migration and development of thymocytes.

In the course of DP cell migration, positively selected cells evolve to CD4SP or CD8SP thymocytes and continue migration towards the medulla, as shown in Figures 2A–B, at ∼10 and ∼170 days respectively. Ultimately, positively selected SP cells in the medulla leave the thymus following S1P gradients, seen in Figure 1C. After ∼100 days of simulation, the system remains virtually unchanged, characterizing a steady state. In the beginning of simulation, DP thymocytes do not undergo apoptosis by lack of stimulus, and are differentiated in SP CD4 or SP CD8, moment where there is a peak of single positive thymocytes around of day 13 (Fig. 2C). At the beginning of simulation the thymocytes DP have a great stimulus from epithelial network, the spatial competition is rather small (“empty” thymus), and the time T_ap_ = 5.6 days required for apoptosis in the model has not yet been achieved. When DP cells initiate apoptosis (15% of DP cells/day), around day 9, the number of single positive thymocytes decrease and stabilizes (Fig. 2D).Interestingly, *in silico* changes in degradation of chemokines resulted in changes of thymocyte migration pattern, increasing the value of *d*, decreasing the chemokine field, and promoting an essentially random thymocyte movement (data not shown).

It should be pointed out that the population of the thymocytes in each developmental stage is strikingly similar to that observed experimentally, the average total number of thymocyte obtained from the mathematical model is approximately 8587.2 cells, where 5% of the thymocytes are in the DN stage, 80% in the DP stage, and 15% in the SP stage [3], while only 1–3% of the thymocytes leave the thymus [25]. In the model presented herein, after the steady state is reached, we found the sub-population rates: 5.3% (451.4±42.3 cells) of DN cells, 79.8% (6848.0±136.3 cells) of DP cells, 14.9% (871.3±27.8 CD4SP cells and 416.5±11.7 CD8SP cells) of SP cells, and only 1.3% (115.6±6.2 cells) of the cells are selected and leave the lattice (Fig. 2D).

Although the mean total thymocyte numbers obtained from the mathematical model (∼8587.2 cells) is smaller than the number of cells experimentally observed (160–320 million cells) [24] and measured with other mathematical models [15] it is important to remark that these measurements are related to the whole volume of the thymus. Here we were interested in relative cell numbers (percentages), since we worked with two-dimensional sections of thymic lobules (the basic physiological unit of the organ). Indeed, dividing the total number of thymocytes found in the lattice by the total number of thymocytes measured in the thymus, we reached the notion that we were working with a very small percentage of the whole thymus (0.0027–0.0054%). It is interesting to observe that this percentage of the whole thymus volume can be obtained directly from our model. In fact and as usually in any two-dimensional model, the basic assumption is such that in the direction perpendicular to the domain in which the problem is defined the behavior is similar. In other words the two-dimensional model is per “unit of depth”. In the case of our mathematical model, the “unit of depth” is 0.0041 mm which multiplied by the area (0.647 mm×0.88 mm) of the cross section used (see the Material and Methods section) gives a volume of ∼0.0023 mm^3^, that corresponds to ∼0.0042% of the normal adult mouse thymus volume: ∼55 mm^3^, as assessed by nuclear resonance imaging and volume displacement [26]. Furthermore, from this percentage (0.0042%) given directly by our mathematical model the number of cells in the mice thymus predicted by the model is trivially obtained and is given by a population of ∼204 million cells (8587.2×100/0.0042) which is in the range of population observed experimentally (160–320 million cells), which in our opinion corresponds to a further validation of the model.

**Figure 3:**
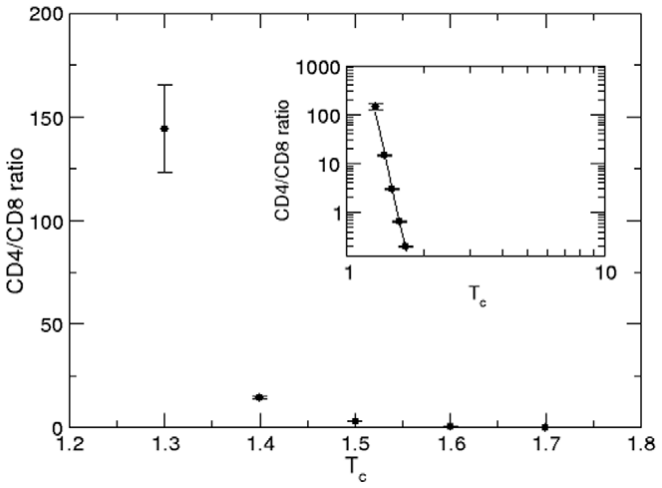
Ratio CD4∶CD8. Ratio between CD4SP and CD8SP thymocytes as a function of T_c_. In the insert, the power law fit has exponent a = −23.3.

In the present model, the CD4SP/CD8SP thymocyte ratio is controlled by the *T_c_* parameter, which defines the average number of successive *T_int_* interactions of a given DP cell with the same epithelial cell needed for differentiation into CD4SP thymocytes or, otherwise in the CD8SP cell subset. The CD4/CD8 ratio increases with time after the fifth day (when the first SP thymocytes appear), from zero to reach a peak around 3 next two weeks of simulation (∼15 days). From this point on it decays, reaching a stationary value around 2, after 70 days of simulation (Figs. 2C–D). This behavior of CD4/CD8 ratio in the early simulation is related with the accessibility to epithelial cells, differing in the empty thymus (early development), as compared to the steady state (filled thymus). In the case of the mouse epithelial network shown in Figure 1A, *T_c_* = 1.53 gives the CD4/CD8SP ratio of 2∶1 (2.09±0.06) (Fig. 3). Of note, the CD4/CD8SP ratio decays very fast with *T_c_*, as seen by the high exponent of the power law. In our model, the number of successive interactions between a thymocyte and a given epithelial cell (thus in the same site) depends on several factors, such as the orientation of the thymocyte during migration, the spatial distribution of the epithelial cell in the neighborhood of that particular thymocyte, the number of thymocytes and their spatial distribution in the neighborhood, etc.

### Permanence Time of Thymocytes in Each CD4/CD8-Defined Developmental Stage

We also verified in the mathematical model presented herein that the time of permanence of each thymocyte in the lattice is very close to that observed biologically [2], as shown in Table 2, thus corresponding to a further validation of the model. Of note, the results are also similar to those generated using another mathematical model based on differential equations [15]. Nevertheless, despite thymocyte velocity being the same in each stage of development, the different times of permanence emerge naturally from the rules established in our model. Instead in the mathematical model using ODE [27], it is necessary to introduce time delay in the equations to simulate the different permanence times of thymocyte subsets.

**Table 2:** Time of permanence for each thymocyte subset in the lattice.

CD4/CD8-defined thymocyte subset	Time of permanence within the organ	
	*Prediction from the mathematical model*	*Experimental Results* *
Double Negative	∼15.83 days	∼15 days
Double Positive	∼6.07 days	∼6.5–9 days
Single Positive	∼5.07 days	∼7–10 days
Total time	∼25.14 days	∼25–30 days

* The experimental results were obtained from [2].

**Figure 4:**
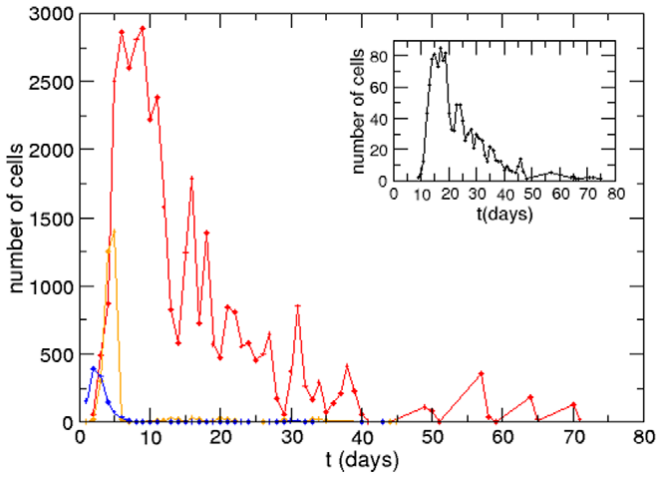
Distribution of permanence time for each thymocyte subset. Mathematically-defined distribution of the permanence time for each thymocyte developmental stage. The insert shows the distribution for the total time of permanence in the thymus, from the entrance of bone marrow-derived precursors to the exit of positively-selected mature thymocytes. Legend of colored lines: red for double negative, orange for double positive cells and blue for mature single positive thymocytes.

The distribution of permanence time for the total thymocytes and for each differentiation stage (DN, DP, and SP cells) can be seen in Figure 4. All distributions are concentrated close to the respective average times, but there are times of permanence that can reach months, as seen in DN cells. These results indicate that the process of maturation and selection in the thymus may involve long periods of time.

### Thymocyte Repopulation After Mouse Sub-Lethal Irradiation

**Figure 5:**
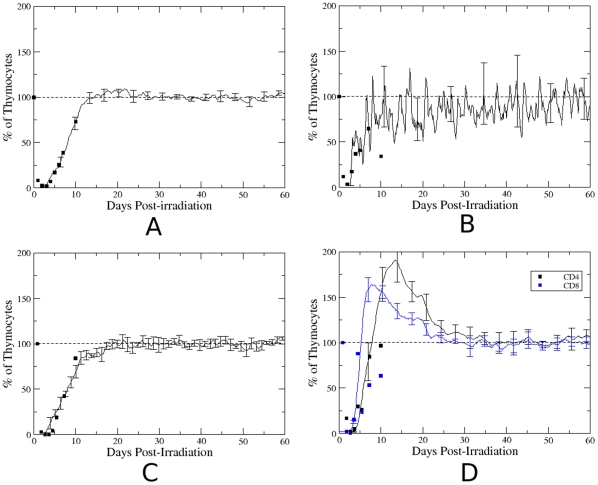
Thymus repopulation. Temporal evolution of percentage of distinct thymocyte subsets in the lattice after the simulation of an irradiation procedure, inserted in the mathematical model. (A) all thymocytes, (B) DN thymocytes, (C) DP thymocytes and (D) SP thymocytes. The symbols represent the experimental values (from ref. [24]) and the solid line represents the mathematical model.

The results of our mathematical model are in agreement with other experimental results and reproduces experiments of mouse sub-lethal irradiation [24]. In this study, mice were irradiated with an A250-cGy whole-body *γ*-radiation dose to induce thymus regression and to study the expression and function of extracellular matrix receptors in distinct thymocyte subsets emerging during the short-term autoctonous repopulation of the organ. As shown in Figure 5, the percentage of cells (measured in relation to pre-irradiation) recovered from thymus rapidly decreased in the first 3 days after irradiation, with minimum values of 1–2%. Thereafter, repopulation started, with recovering to the normal values 10 days post-irradiation [24]. Using our mathematical model we also evaluated thymocyte repopulation in the lattice, simulating the irradiation experiment. Simulation was done by elimination of 98% of the thymocytes after having reached the steady state. Different from what occurred experimentally, in which the thymocyte died rather slowly due to the irradiation, in the mathematical model they are eliminated instantly from the lattice. Then for purposes of comparison, the results of our mathematical model were confronted to the experimental data obtained after the 3^rd^ day of post-irradiation, when the repopulation of the mouse thymus starts. As seen in Figure 5A, the mathematical model perfectly reproduces the rate of repopulation of the thymus for all thymocyte subsets. In the same Figure, we can also observe that the mathematical curve tends to saturate after the ∼10 days of simulation, which is the time during which the population of mouse thymocytes returns to normal. The large fluctuation of temporal evolution of relative DN cell numbers (Fig. 5B) compared with the other thymocyte subsets, is due to the explosion of divisions that occurs when a single DN cell reaches the subcapsular zone, a region of intense proliferation. Interestingly, the repopulation of CD8SP thymocytes occurs faster than that of CD4SP cells (Fig. 5D), which may indicate an advantage of the CD8SP over CD4SP thymocytes when cells are in abnormal concentrations. By contrast, the DP thymocyte subset (see Fig. 5C) is similar to the profile of the total repopulating cells (Fig. 5A), which is in keeping with the fact that DP cells represent the vast majority of thymocytes.

In these simulations, the relative numbers of CD4 and CD8 SP thymocytes increave above the normal values. This is likely explained by the fact that at the beginning of simulation (first week), the spatial competition between thymocytes is relatively small and DP thymocytes have not undergone apoptosis by lack of interaction with the epithelial profile, then there is the overshoot of single positive thymocytes. After the first week of simulation, the great number of thymocytes in the lattice increase the spatial competition and after a time Tap, DP cells can die by apoptosis by lack of interaction with the epithelial profile (15% of DP cells/day), then the number of single positive thymocytes decrease and stabilizes thereafter.

### Similar Mathematical Behavior of Thymocytes in a Human Thymic Epithelial Network

**Figure 6:**
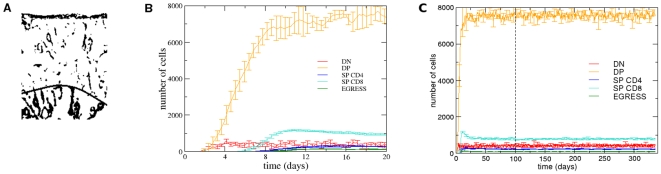
Human thymic epithelial network and corresponding temporal evolution of thymocytes. The epithelial network shown in Panel (A) derives from immunohistochemistry of a 2 years-old infant thymus section. Panel (B) and (C) depicts the mathematically-defined temporal evolution of thymocytes in each developmental stage on early stages and in the steady state respectively. The vertical dashed line indicates the moment at which the system reaches a steady state. Note that the temporal evolution of thymocytes in each developmental stage is similar to that elicited in the normal mouse thymic network seen in Figure 2C and 2D.

To compare the results obtained with the mouse epithelial network, we also evaluated the dynamics of the model with a human epithelial network derived from data obtained experimentally in our laboratory, by immunolabeling a histological section of a normal child thymic tissue with a pan-cytokeratin antibody. In this epithelial network we observed the same density of occupation, as depicted in Figure 6.

The temporal evolution of thymocytes obtained with the human epithelial network, Fig. 6B and 6C exhibited similar behavior of those in the mouse thymic epithelium (Fig. 2C and 2D) (see the Movie S2). When we used the same value to the parameters studied herein, the populations of thymocytes asymptotically achieved a steady state, as seen in Figure 6C. The mean total thymocyte numbers obtained from the mathematical model using the human epithelial network is approximately 9000 cells. The number of cells in each thymocyte subset is closer to that in the mouse epithelial network: 4.7% (419.8±42.4 cells) of DN cells, 84% (7559.4±128.8 cells) of DP cells, 11.4% (230.4±8.75 CD4SP cells and 789.3±16.0 CD8SP cells) of SP cells, and 0.9% (83.9±4.35 cells) of cells that are selected and leave the lattice. The CD4SP over CD8SP cell ratio as a function of *T_c_* also follows a decay in a power law, as we found using the mouse epithelial network, but with the exponent *a* = *−*20.5. A 2∶1 ratio using the human epithelial network was achieved for *T_c_* = 1.39, thus smaller than that obtained with the mouse network.

Overall, the results derived from the mathematical model indicate that the thymus architecture (namely the spatial distribution of the epithelial network) may also be important to thymocyte lineage commitment and may influence the CD4/CD8 cell ratio.

### Biologically-Structured Epithelial Cell Network Is Necessary for a Normal Thymocyte Development, as Defined by the Cellular Automata Lattice

**Figure 7:**
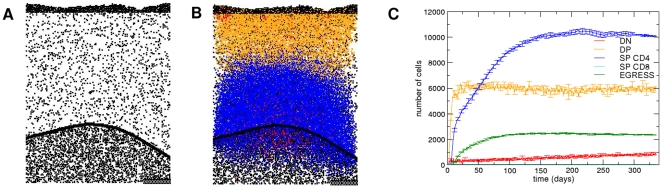
Theoretical behavior of thymocytes in a mathematically-generated epithelial network: temporal evolution and lattice distribution. Panel (A) shows a different profile, generated randomly that do not match with panels of Figures 1A and 6A. Panel (B) reveals the abnormal spatial distribution of thymocytes upon interaction with a random epithelial network, as seen at 335 days of simulation. Additionally, as seen in panel (C), the temporal evolution of thymocytes in each developmental stage within the mathematically-generated network differs from that elicited in the normal mouse or human thymic networks, seen in Figures 2D and 6C, respectively. Legend of colors: red for the DN cells, orange for the DP cells, blue for the CD4SP cells, cyan for the CD8SP cells, and green for the number of cells that leave the thymus.

We further evaluated the putative influence of the thymus architecture using an epithelial network that was generated randomly, as shown in Figure 7. Each microenvironmental niche of the random network had the same density of occupation, as compared with the mouse network, but the positions of the overlapped sites were changed randomly. In this case the spatial distribution of the mathematical epithelial network was more uniform and did not present the large clusters seen in biological epithelial networks. Figure 7 shows that the temporal evolution of the thymocyte subsets upon interaction with the random epithelial network assume different dynamics, when compared with the results of interactions with the mouse and human epithelial networks (seen in Figs. 2D and 6C, respectively). Within the random network, thymocyte subpopulations did not reach a steady state and increased with time and saturated the lattice, especially with CD4SP thymocytes (see the Movie S3). Interestingly, in this random network, the rate of cells leaving the thymus was smaller than the numbers of positively-selected SP thymocytes, resulting in an increase of cell numbers with time, seen as large numbers of CD4SP cells (Figs. 7B–C).

After 200 days of simulation, the average number of thymocytes in the lattice was around 16950 cells, two times the number of cells obtained with the mouse epithelial network. The large amount of thymocytes in the lattice can be explained by the absence of large clusters of the epithelial network, which increase the availability of epithelial cells to interact with thymocytes and decrease the apoptosis by lack of stimulus. Interestingly if at day 50 of simulation is reestablished the normal mouse epithelial network, the selection process and proportions of thymocyte populations normalize after seven weeks.

The small number of CD8SP thymocytes in the lattice (∼28 cells) can also be explained by the absence of large clusters of epithelial cells, with a consequent increase in the probability of a given DP thymocyte to interact with the same site in its neighborhood and to promote the differentiation into CD4SP thymocytes.

## Discussion

The mathematical model herein developed for thymocyte migration and differentiation is in agreement with experiments that evaluated thymus repopulation from double-negative cells, following sub-lethal irradiation in mice [24]. Compared with other mathematical models previously published, especially those exploring the dynamics of thymocyte subsets [12], [14], [15], this is the first mathematical approach that describes DN→DP→SP thymocyte development in the context of interactions with the thymic epithelial network, whose positions in the lattice were extracted from experimental data of immunohistochemistry performed in mouse or human thymus sections. With this strategy it is possible to study and to compare these results with other epithelial networks, including those derived from pathological situations in which the thymic architecture was modified as described in autoimmune and infectious diseases. In this respect, we showed that simulations made using a mathematically-devised random epithelial network presented a specific dynamics that differed from those observed for the experimental mouse or human epithelial networks.

In our mathematical model, we also approached the CD4 *versus* CD8 lineage commitment. This can be determined by the duration of the TCR signal: Long duration signals favor development of CD4SP cells and short duration signals rather favor differentiation into CD8SP cells [8]. The results shown herein indicate that the thymus architecture is important in determination of the duration of the TCR signal; simulations performed with mouse and human epithelial networks provide different CD4∶CD8 ratios, using the same set of parameters. These lineage commitment results are at apparent variance with those reported by Efroni and co-workers [14], who showed that the thymocyte fate at a 2∶1 CD4∶CD8 ratio may be determined by the thymocyte dissociation rate with the epithelial cells, which is lower for CD8 than for CD4 thymocytes. However, it is conceivable that such thymocyte dissociation from the thymic epithelium precisely derives from changes in the epithelial network.

Since the simulations were performed using a 2-D lattice, the results derived from a 3-dimensional version of the model may present some differences in respect to thymocyte moving interactions and divisions. Considering that a 3-D sponge-like epithelial network has one more degree of freedom, we could expect changes in the thymocyte spatial competition and in CD4/CD8 ratio. Nevertheless, it its worthy to point out that the final numbers of thymocytes obtained when we took into account the whole volume of the organ, fell within the range actually seen in the thymus [24], thus further validating the model.

Previous studies showed that chemokines produced by thymic microenvironmental cells, together with extracellular matrix proteins (also secreted by the thymic microenvironment), have a pivotal role in thymocyte migration, and data suggest a combinatorial role for these molecules in this migratory process [3], [28], [29]. Accordingly, the appropriate migration in each thymocyte developmental stage is driven by simultaneous and/or sequential *stimuli*, and this is important for normal T cell differentiation. More recently, we further developed this concept, proposing that thymocyte migration is actually derived by a resulting migration vector, which in turn appears as a consequence of various migration vectors (each one corresponding to a given ligand/receptor pair interaction) that can differentially contribute to generating the resulting migration vector [29], [30]. In this context, future development of the current mathematical model and the subsequent implementation of new rules, including other cell migration-related molecules, will allow a more complete mathematical description of the migratory phenomena. With this, we will have an important mathematical tools to predict changes in thymocyte migration and even to simulate therapeutic strategies having thymocyte migration as biological targets.

In any case, the cellular automata-based mathematical model for thymocyte differentiation and migration could be useful in evaluating abnormal thymocyte differentiation/migration and in predicting thymocyte evolution following therapeutic strategies designed to recover the thymus in diseases coursing with thymus involution, such as some primary immunodeficiencies, acute infections, and malnutrition [15], [31].

## Materials and Methods

The computational language used in the mathematical model implementation was FORTRAN 90 (http://www.intel.com) (see the basic program on File S1), in its noncommercial free version for LINUX. To generate the graphics we use the free software library G2 (http://g2.sourceforge.lattice), labplot (http://labplot.sourceforge.lattice/) and the xmgrace (http://plasma-gate.weizmann.ac.il/Grace).

**Figure 8:**
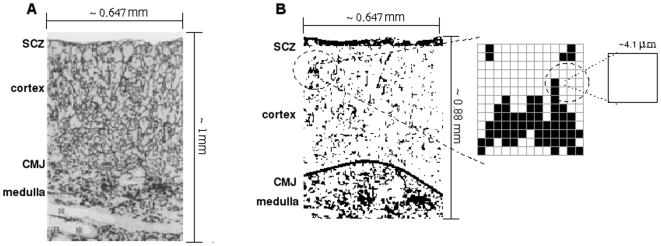
Lattice and mouse thymic epithelial cell network. (A) A histological section of a mouse thymus showing the epithelial network, as derived from the work by van Ewijk et al., 1999 [33]. The epithelial network was used in the model for interacting with and allowing migration of thymocytes. Panel (B) reveals the details of the epithelial network showing the sites of the lattice. Each site has approximately 4.1 µm×4.1 µm. The total lattice has 196×158 = 30968 sites. SCZ  =  subcapsular zone, CMJ  =  cortex-medulla junction.

The simulations were performed using a two-dimensional lattice representing a section of the thymic lobule (cortex and medulla) derived from actual C57BL/6 mouse thymus histological cross section as seen in Figure 8. This two-dimensional lattice was partitioned in H = *196×L* = 158 = 30968 sites, each site has ∼16* µm* ^2^, which is approximately the thymocyte dimension [32]. This can be seen in Figure 8B, where we can also observe four important microenvironments of the thymic lobule for thymocyte migration and maturation: subcapsular zone (SCZ), cortex, cortex-medulla junction (CMJ) and medulla. Furthermore, to represent the normal thymus architecture the density of the epithelial profile in Figure 8B was adapted in each microenvironment so that ∼80% in the SCZ, ∼7% in the cortex and ∼30% in the medulla [2]. In the model, each site represents a given thymocyte in a given CD4/CD8-defined developing stage: double-negative, double-positive or single-positive, which can move in the lattice following spatial gradients of chemokines, can proliferate, can interact with the epithelial microenvironment, and can die by apoptosis. Thymocyte interactions with an epithelial profile that was overlapped in the lattice (and obtained from experimental results with mice [33]) trigger signals to cell differentiation, as depicted in Figure 8B. To this end, to each site *i* overlapped by the epithelial network is assigned a random variable *APC*(*i*) = [0, 1) that represents the MHC/peptide of the microenvironmental cell that will interact with the thymocyte, as explained below in double-positive cell rules. Finally, the lattice update was made in parallel, whereas all sites were updated simultaneously.

### Spatial Rules for Movement

The thymocyte moves in each lattice update with speed *S* and its migration is influenced by gradients of concentration created by the diffusion of different chemokines produced by the epithelial network. Specific sites overlapped by the epithelial network produce *K* amounts of chemokine in each lattice update. The thymocyte moves preferentially towards the site of its neighborhood in which the concentration of the chemokines is higher. In this case, the site is chosen to move with probability:
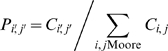
(1)


Where *Ci′,j′* represents the concentration of the chemokines in the site (*i′, j′*), the sum run over the Moore's neighborhood of (*i, j*) (first and second neighbors), the actual site of the thymocyte. If another thymocyte exists in the site (*i′, j′*) chosen for the movement, the thymocyte stays immobile in the site (*i, j*) until the next lattice update, when then again a new site (*i′, j′*) is chosen for the movement. Which chemokine source will guide the thymocyte movement depends on the developmental stage of each cell.

Along with thymocyte migration, if the chosen site for migration is overlaid by the epithelial network, an additional chance *p_mov_* will be launched. Thus, the migratory movement in the two-dimensional lattice is preferentially between the sites overlapped by epithelial profiles.

The diffusion of the chemokine is obtained using the Laplace's equation (*∇* ^2^ = 0) solved by finite differences [34], as follows:
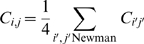
(2)


The sum run over the Newman's neighborhood of site (*i, j*) (first neighbors). The chemokines in each lattice updating also undergo enzymatic cleavage [28], losing a fraction *d* of their concentration in each site. Since in this mathematical model the chemokine sources do not change, the values adopted for the concentration to each chemokine in the lattice are those reached in the steady state. The rules for emigration and for each thymocyte stage in the model are described below. Should we state that, after pre-designing this mathematical model, we checked whether it would fit experimental data on thymus replenishment following sublethal γ-ray radiation [24]. Values of some parameters were then adjusted, so that to fit with the thymocyte recovery seen in these *in vivo* conditions.

### Double-Negative Thymocytes

The DN cells enter the lattice by sites of the cortex-medulla junction [2], [24] chosen randomly at a *T_entry_* rate. The migration of the DN cells towards the SCZ follows the CXCL12 chemokine gradient, whose larger concentration is located in the subcapsular zone [4], [35], [36]. In the lattice, only sites bearing epithelial cells overlapped in the SCZ produce CXCL12. Accordingly, the introductory model only considers chemotaxis driven by CXCL12.

In the SCZ, double-negative cells start the replication process at a *T_div-DN_* rate, the division generating two cells with similar characteristics. One of the cells moves to one of the sites of Moore's neighborhood of the mother cell. Migration occurs towards the thymocyte-free empty neighboring sites with equal probability. If no empty sites are available, the cell division occurs in the next lattice update in which empty neighboring sites occur. Each double-negative that enters the lattice can originate *N_max_* descendants.

In the SCZ, the double-negative cells differentiate in double-positive thymocyte cells at a *T_DP_* rate. Despite the fact that our model does not consider developmental stages for each thymocyte subset, we apply the spatial information to differentiate these cells.

### Double-Positive Thymocytes

In the subcapsular zone, CD4^+^CD8^+^ thymocytes proliferate at the *T_div-DP_* rate. Migration of these cells occurs towards the medulla, attracted by the CCL19 and CCL21 chemokine gradients [37], [38]. For simplicity, the sites overlapped by the epithelial network in the medulla, which produces the chemokines CCL19 and CCL21, are represented in the introductory model as one gradient. In our model no distinction is made between these two chemokines. In other words, the sites overlapped by the epithelial network in the medulla produce CCL19/CCL21 chemokines and the migration is governed by the corresponding concentration gradient.

To each new DP cell, a random variable *TCR*  =  [0, 1) is chosen, representing its TCR receptor in the interaction with the epithelial network. Positive selection or apoptosis of a given DP cell will take place when:
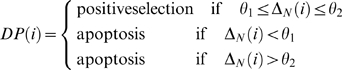
(3)


Where, 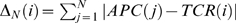 measured after *N = N_DP_* interactions of the DP cell (*i*) with the sites overlapped by epithelial network, occurring during its migratory process.

The interactions between a given DP thymocyte and the epithelial network occurs in its own site (*i*) or in Newman's neighborhood, and have a duration time of *T_int_*. After the *T_int_*, the DP cell that had stopped, starts the migratory process again, allowing to occur new interactions with the epithelial network, including the very last one.

If Δ*_N_*(*i*) is smaller than *θ*_1_, high affinity exists between the DP cell TCR(*i*) receptor and the MHC/peptide of the corresponding epithelial cell, resulting in thymocyte death by apoptosis. If Δ*_N_*(*i*) is larger than *θ_2_*, a low affinity exists between the DP cell TCR(*i)* receptor and the MHC/peptide of the epithelial cell, also resulting in the thymocyte death by apoptosis. The positive selection and differentiation in the SP cell occurs when the DP cell TCR(*i*) receptor interacts with an intermediate affinity. We can also identify Δ*_N_*(*i*) as avidity. Indeed, the TCR is probed with *N* molecules MHC plus peptide. In the present model, if the DP cell does not succeed in interacting with *N* sites overlapped by the epithelial network before a time *T_ap_*, then the thymocyte will also die by apoptosis due to lack of *stimuli*.

Biologically, if the TCR has a very low affinity with the MHC/peptide complex, the thymocyte dies by apoptosis, due to lack of interaction. Similarly, if the TCR has a high affinity with the MHC/peptide complex, the thymocyte also dies by apoptosis, and this largely prevents the development of auto-reactive T cell clones, and thus autoimmune diseases. We should point out that, despite the fact that the generation of TCR specificities involves a complex process gene rearrangement [1], in our initial model we are mathematically considering the generation of TCR specificities as a random process.

### Single Positive Cells

In the present model we consider that the duration of the TCR-induced signal determines the lineage commitment of each given T cell [7], [8], [39]. The signal intensity Δ*_N_* in equation 3 only determines the number of DP cells undergoing positive selection. Those positively-selected DP cells exhibiting a given number of successive interactions *T_int_* with the same site on average larger than *T_c_*, will differentiate in the CD4SP cell and otherwise in the CD8SP cell. The SP cell, either CD4 or CD8, continues the migration towards the medulla, induced by the CCL19 and CCL21 chemokine gradients. Within the medulla SP cells also undergo a selection process after *N_SP_* interaction with epithelial cell profiles, similar to what happens with DP cells (equation 3). This negative event ensures the complete thymocyte development as well as central tolerance.

### Emigration

When studying thymocyte egress, we initially considered that the post-selection SP cells are guided through the S1P gradient and then leave the thymus, crossing the endothelial barrier at the cortex-medulla junction [40]. Biologically, S1P is largely derived from the blood, gaining the blood vessels in the thymus. For simplicity, we considered herein that S1P is produced at the CMJ.

## Supporting Information

Movie S1 Thymocyte migration and development in the mice epithelial network. Computational simulation of thymocyte migration and development in the lattice overlapped by mice epithelial network. Legend of colors: black for the epithelial network, red for DN cells, orange for DP cells, blue for CD4SP cells, and cyan for CD8SP cells. The black color is shown for the site with a thymocyte and a portion of the epithelial network.
(3.46 MB MOV)

Movie S2 Thymocyte migration and development in the human epithelial network. Computational simulation of thymocyte migration and development in the lattice overlapped by human epithelial network. Legend of colors: black for the epithelial network, red for DN cells, orange for DP cells, blue for CD4SP cells, and cyan for CD8SP cells. The black color is shown for the site with a thymocyte and a portion of the epithelial network.
(2.23 MB MOV)

Movie S3 Thymocyte migration and development in a mathematical epithelial network. Computational simulation of thymocyte migration and development in the lattice overlapped by a random epithelial network mathematically generated. Legend of colors: black for the epithelial network, red for DN cells, orange for DP cells, blue for CD4SP cells, and cyan for CD8SP cells. The black color is shown for the site with a thymocyte and a portion of the epithelial network.
(4.20 MB MOV)

File S1 FORTRAN90 PROGRAM
(2.21 MB GZ)
